# Supplementary figures and images for: Reducing options of ammonia volatilization and improving nitrogen use efficiency via organic and inorganic amendments in wheat (Triticum aestivum L.)
Source: PeerJ. 2023 Mar 6;11:e14965. doi: 10.7717/peerj.14965 (PMC9997193; doi:10.7717/peerj.14965)

**
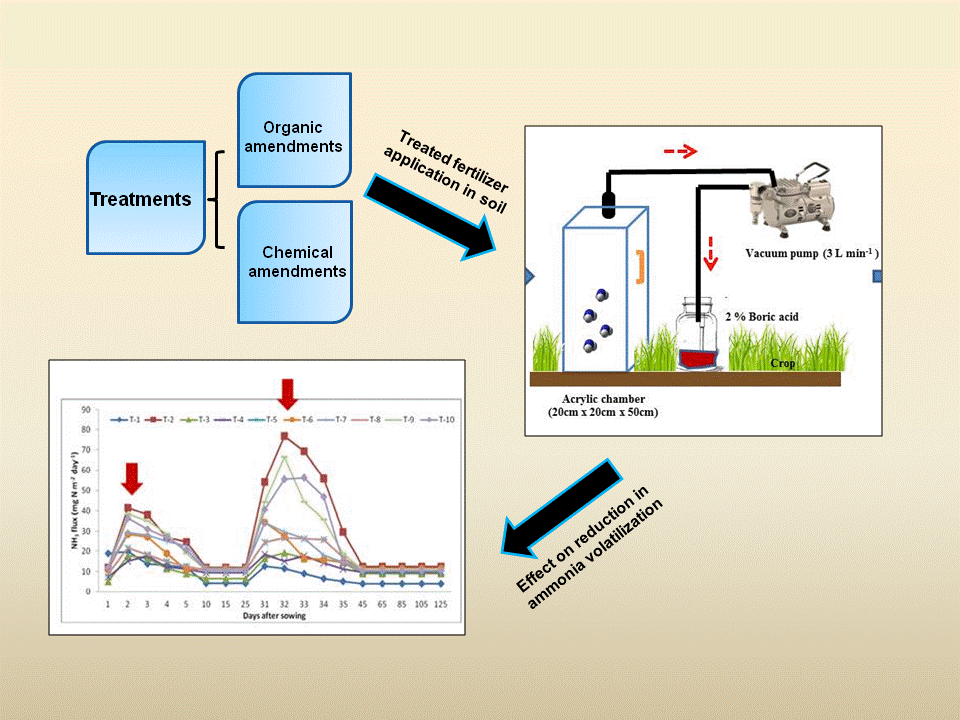
**

Supplement: Supplemental Information 2 [file peerj-11-14965-s002.docx]
